# Supplementary material for: Semiconductor SERS enhancement enabled by oxygen incorporation
Source: Nat Commun. 2017 Dec 8;8:1993. doi: 10.1038/s41467-017-02166-z (PMC5722866; doi:10.1038/s41467-017-02166-z)
Supplement: Supplementary file 1 — Supplementary Information [file 41467_2017_2166_MOESM1_ESM.pdf]

Supplementary Figures

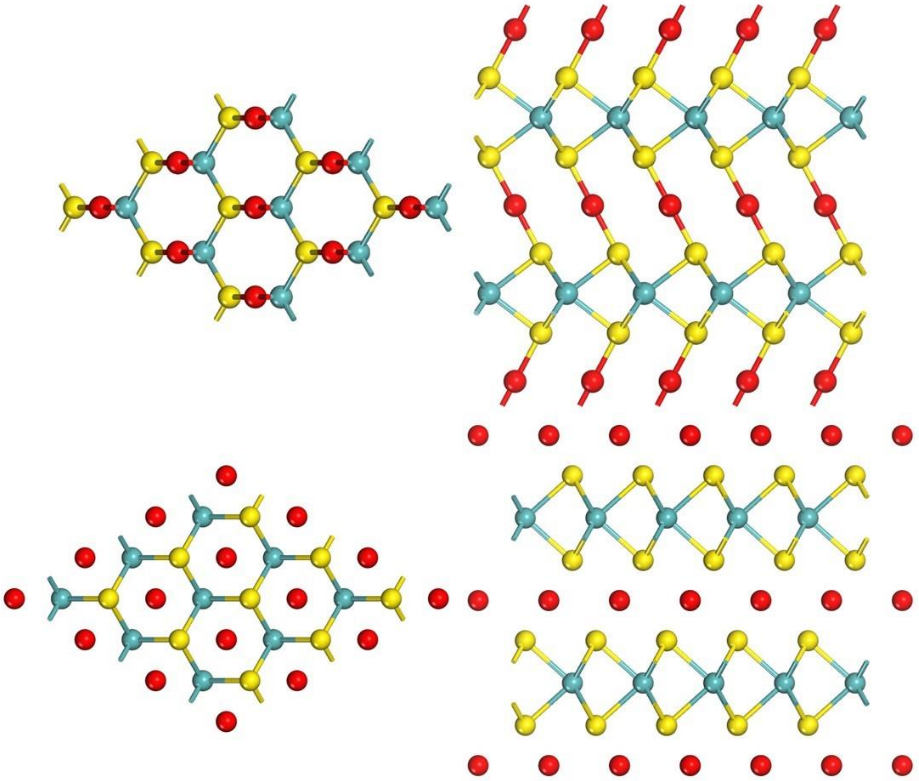

Supplementary Figure 1 Atomic structure of oxygen-incorporated MoS_2: upper panel, bridge configuration; lower panel, hollow configuration.

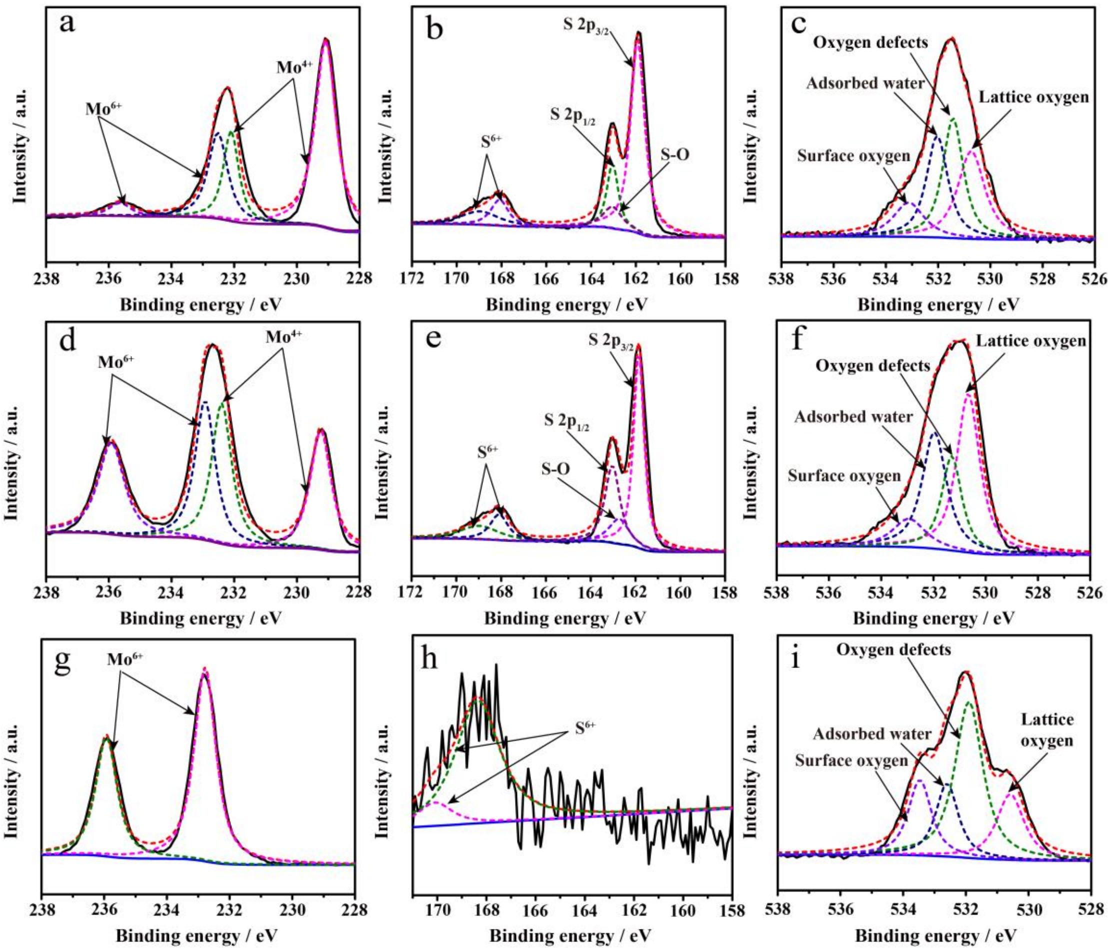

Supplementary Figure 2 XPS spectra showing the binding energies of molybdenum, sulfur, and oxygen. (a-c) MoS_2-250 ℃-30 min, (b-d) MoS_2-300 ℃-30 min (g-i) MoS_2-350 ℃ -30 min.

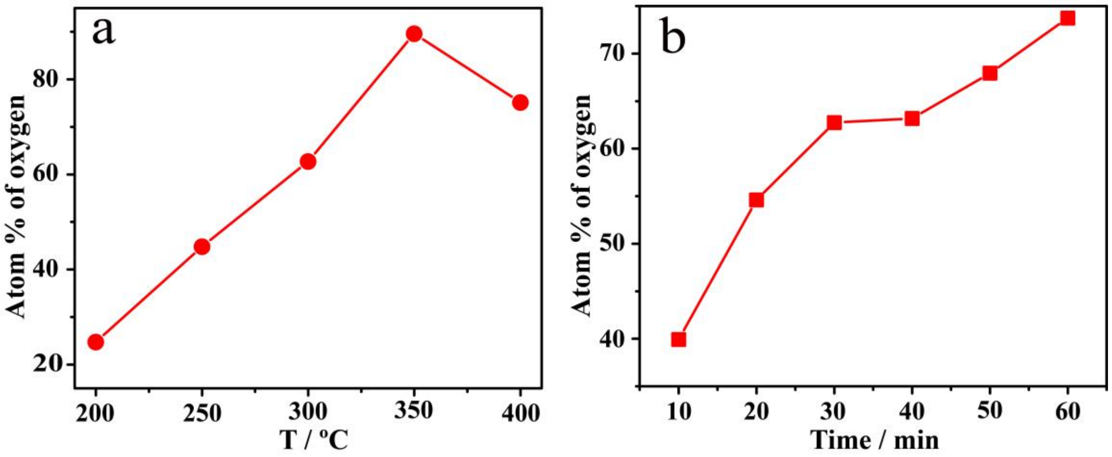

Supplementary Figure 3 The effect of annealing time and temperature on oxygen incorporation concentrations of oxygen-incorporated samples.

a

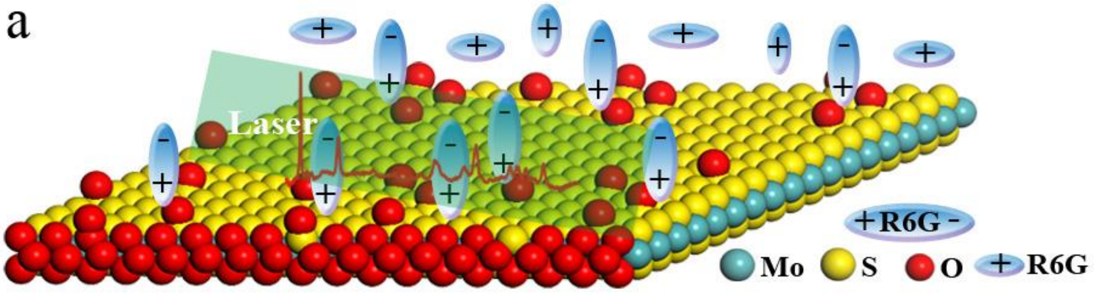

b

Bader charges	Mo ion (e)	S ion (e)	O ion (e)
MoS_2	1.73	-0.86	----
MoS_xO_y	1.60	-0.50	-0.58

Supplementary Figure 4 (a) A schematic diagram illustrating the generation of dangling bonds, which probably weaken the constraint to the surface electrons by a redistribution of the electron density in MoS_2 and then further contribute to the SERS enhancement. (b) Calculated Bader charges of pristine MoS_2 and oxygen-incorporated MoS_2.

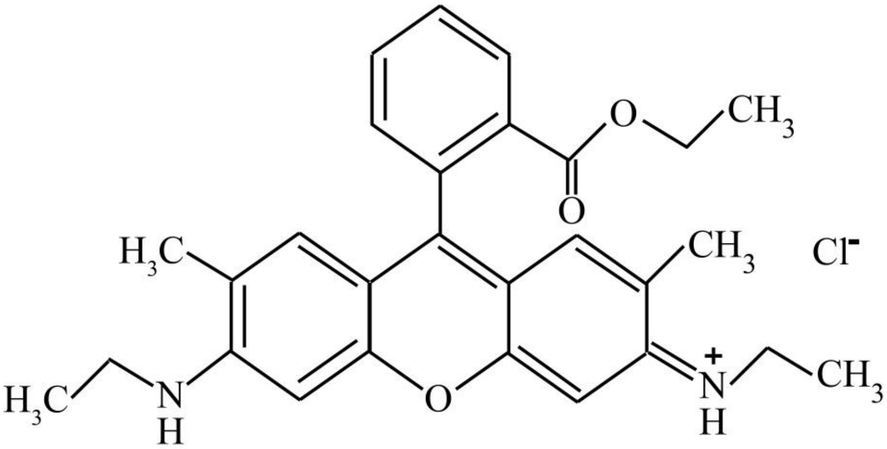

Supplementary Figure 5 Chemical structure of Rhodamine 6G.

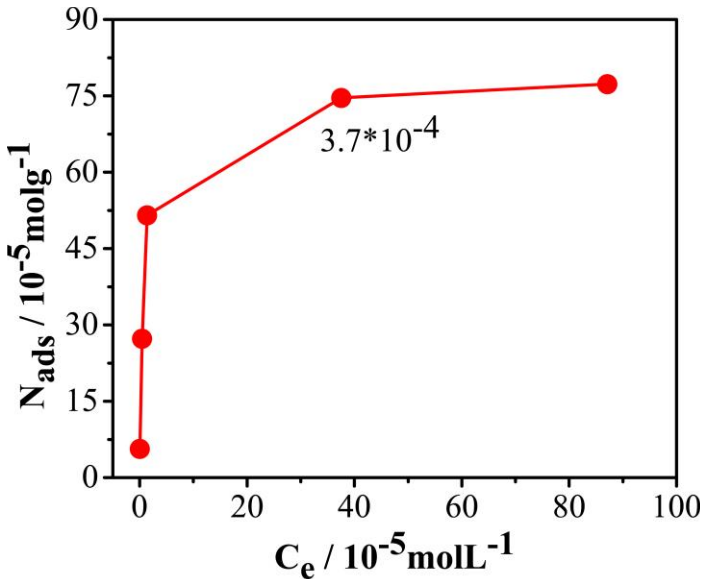

Supplementary Figure 6 R6G adsorption isotherms. Adsorbed amount versus equilibrium concentration of R6G in the presence of as-prepared oxygen-incorporated MoS_2 sample. For an initial concentration of R6G lower than $3.7*10^{-4}$ M, the maximum amount of R6G adsorbed onto oxygen incorporated MoS_2 substrate (effective substance concentration of about 0.5 mg / mL) is not reached.

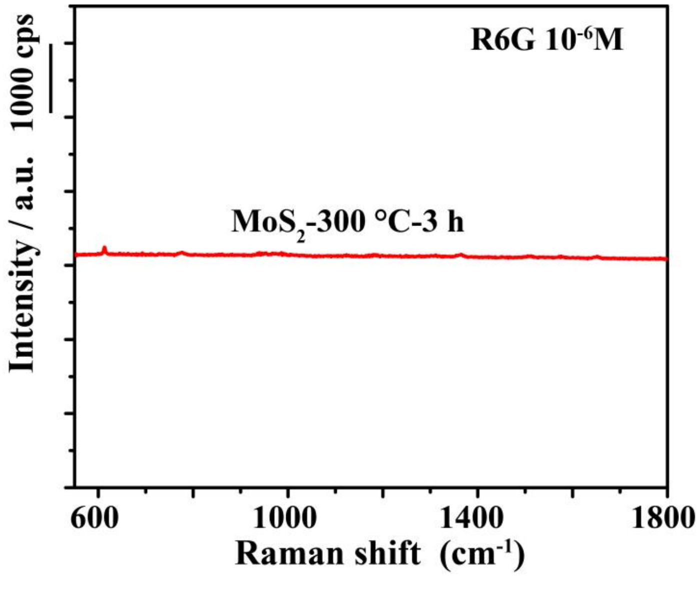

Supplementary Figure 7 SERS spectra of oxygen-incorporated MoS_2 sample annealed at 300 ℃ for 3 h.

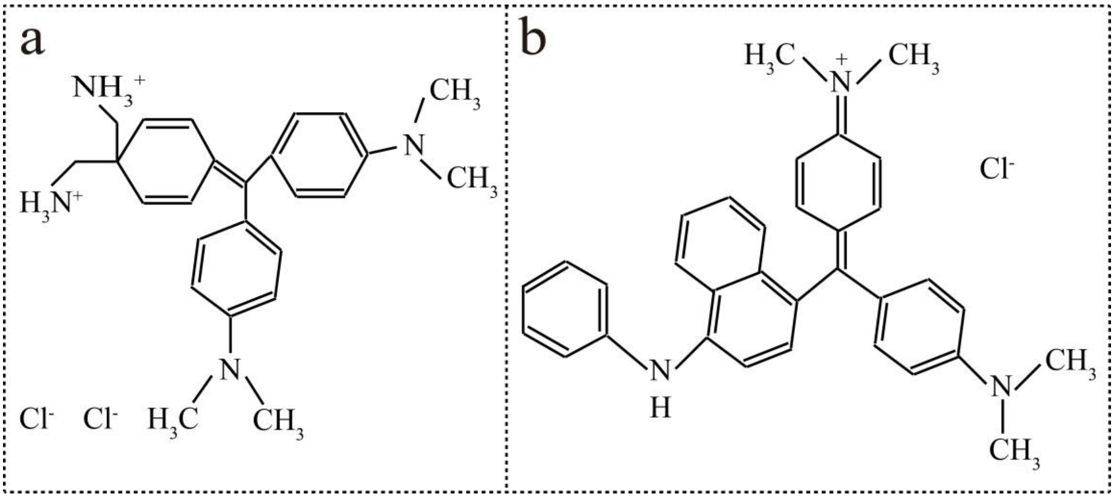

Supplementary Figure 8 Chemical structure of crystal violet (a) and victoria blue B (b).

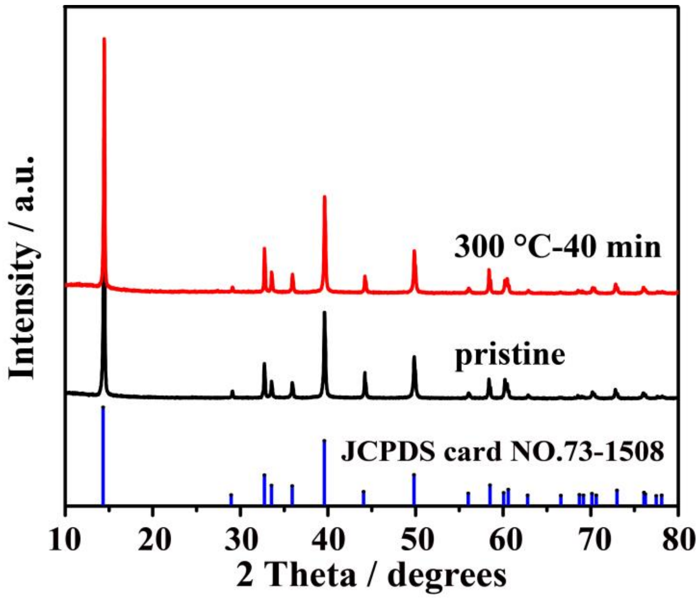

Supplementary Figure 9 XRD pattern of the oxidized sample obtained at 300 ℃ for 40 min, and the standard patterns of the pristine 2H-MoS_2 (JCPDS No.73-1508).

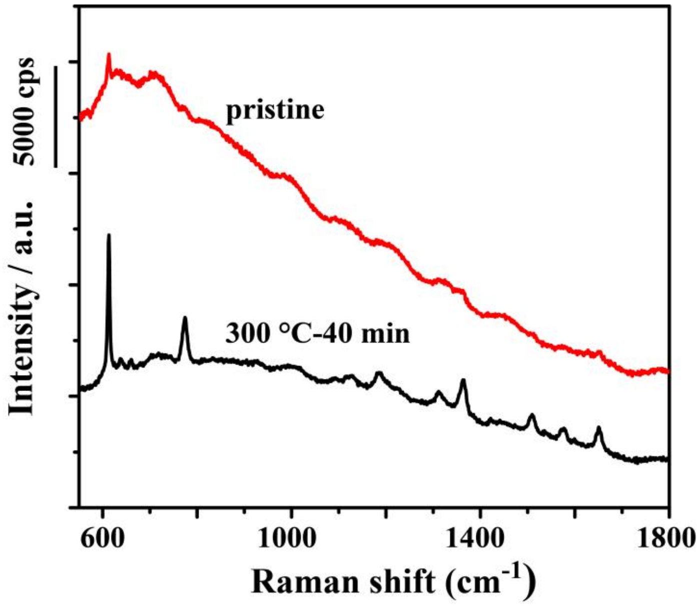

Supplementary Figure 10 SERS spectra of the oxygen-incorporated bulk MoS_2 sample annealed at 300 ℃ for 40 min and pristine bulk MoS_2. It has been found that the bulk MoS_2 still keeps its pristine crystal phase after being annealed at 300 ℃ for 40 min in air, but the Raman signals of R6G can be can be considerably enhanced by oxygen incorporation as well. When the concentration of R6G is 10^{-5} M, the EFs can reach as high as 0.9×10^5. However, the pristine sample only yields very weak R6G Raman signals over the fluorescence band.

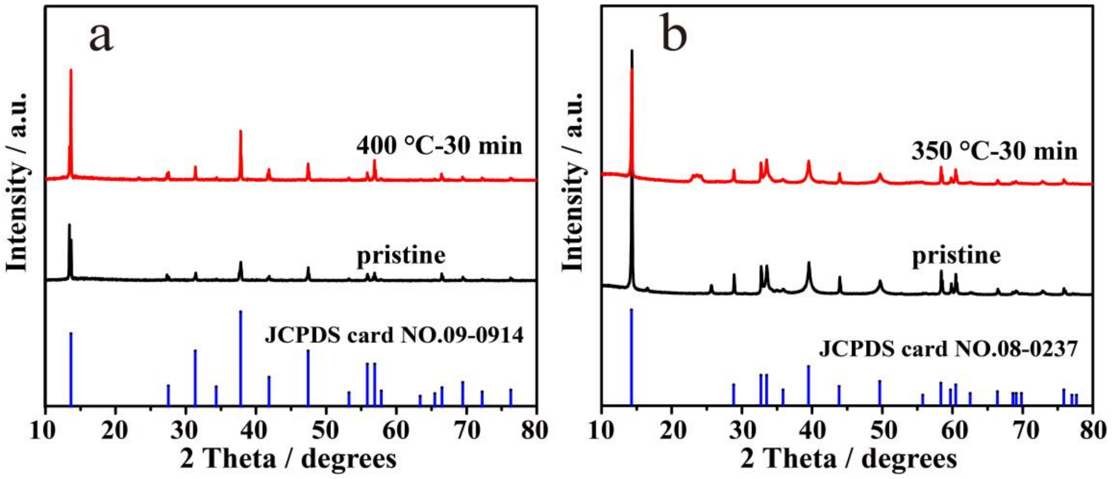

Supplementary Figure 11 XRD patterns of the oxidized $MoSe_2$ and WS_2 samples obtained at various annealing temperatures, and the standard patterns of the pristine 2H-$MoSe_2$ (JCPDS No.09-0914) and 2H-WS_2 (JCPDS No.08-0237).

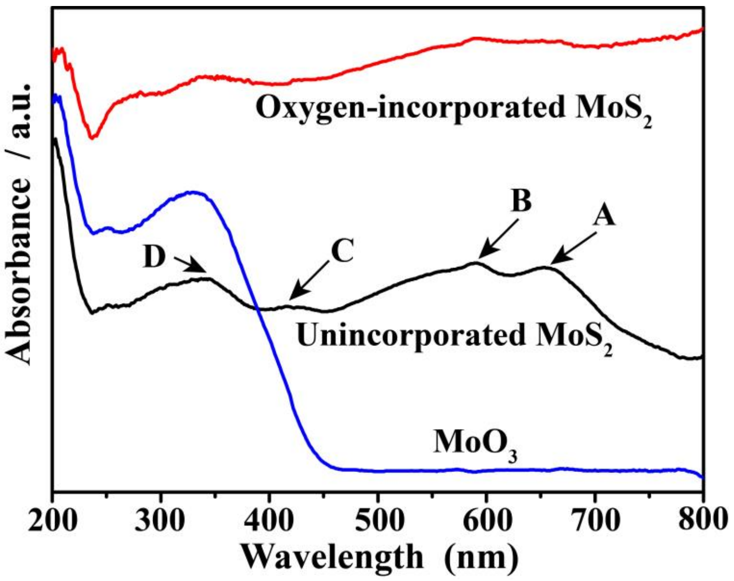

Supplementary Figure 12 UV-Vis absorption spectra of unincorporated MoS_2, oxygen-incorporated MoS_2 and MoO_3.

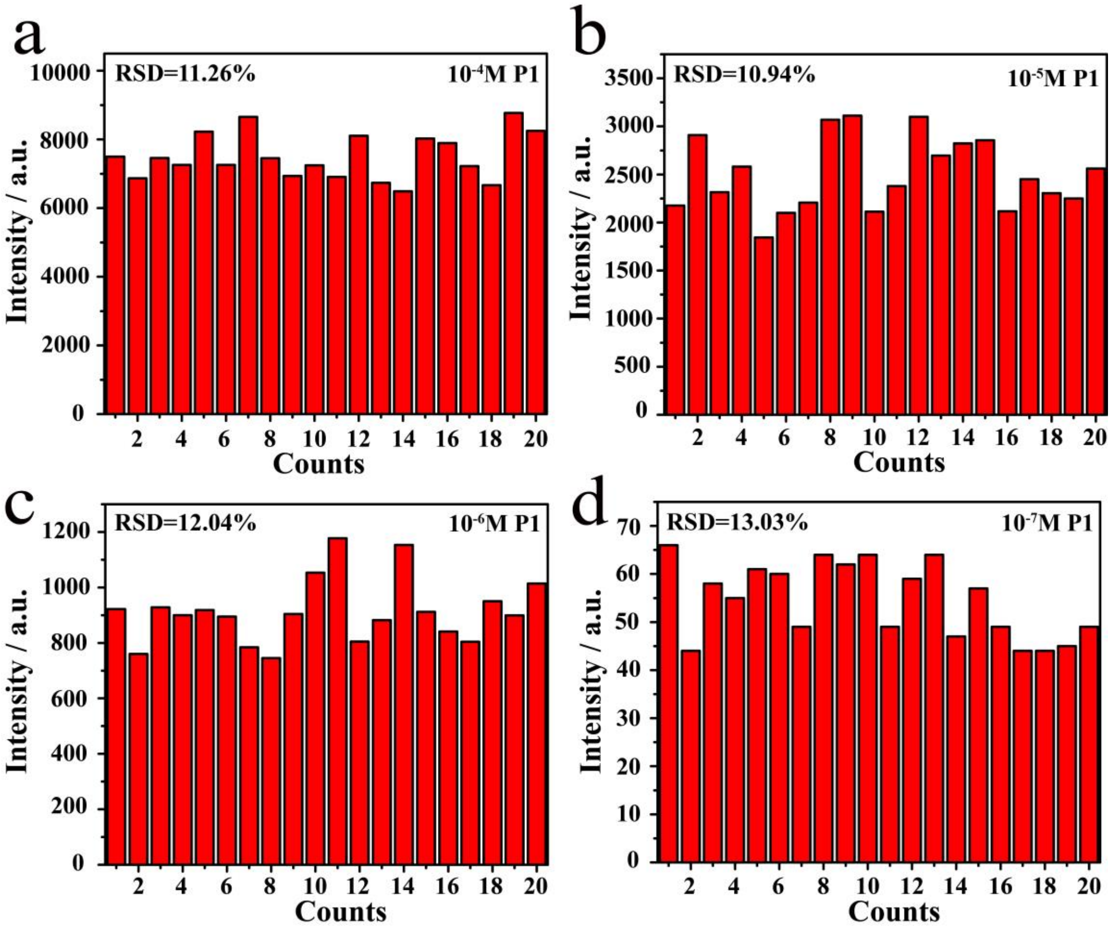

Supplementary Figure 13 The intensities of the P1 (612 cm^{-1}) Raman vibration mode of R6G on varied spots. Four different concentrations, 10^{-4}, 10^{-5}, 10^{-6}, and 10^{-7}

M, were examined with data acquired by recording spectra at 20 stochastic spots at different locations across from one substrate. The RSD of the P1 (612 cm^{-1}) Raman vibration mode of R6G at four different concentrations, 10^{-4}, 10^{-5}, 10^{-6}, and 10^{-7} M, are calculated to be 11.26%, 10.94%, 12.04% and 13.03%, respectively.

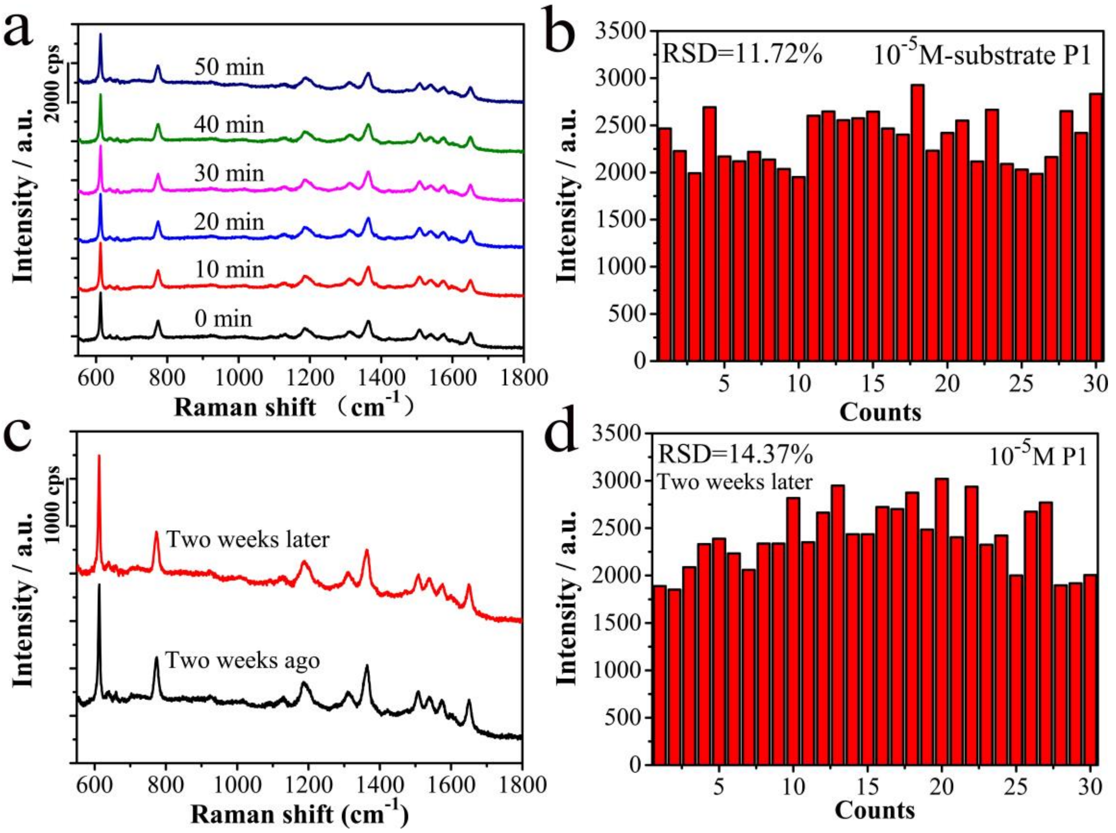

Supplementary Figure 14 Working lifetime of the SERS substrate. SERS performances of the partially-oxidized MoS_2 samples obtained at 300 ℃ for 40 mins are recorded every 10 minutes (a) and before/after two weeks of storage in the open air (c). Reproducibility and stability are two important issues for SERS substrate performance. Firstly, the relative standard deviation (RSD) of the characteristic peak of R6G at 612 cm^{-1} is used to estimate the reproducibility of the SERS signal. (b) shows the SERS-RSD spectra of R6G molecules, randomly collected from 30 positions of six substrates. The RSD value is calculated to be 11.72%, indicating good reproducibility. Secondly, to investigate this stability of substrates, the partially-oxidized samples are kept in in the open air for two weeks and then performed SERS measurement on them. The collected SERS spectra after two weeks

of storage in the open air are compared with those obtained from the freshly prepared substrate (c). It is noted that neither a shift in the major Raman peaks nor a significant change in Raman intensity occurred for two weeks, and the RSD value remains as low as 14.37% (d). The result reveals that the as-prepared substrate is stable for at least two weeks.

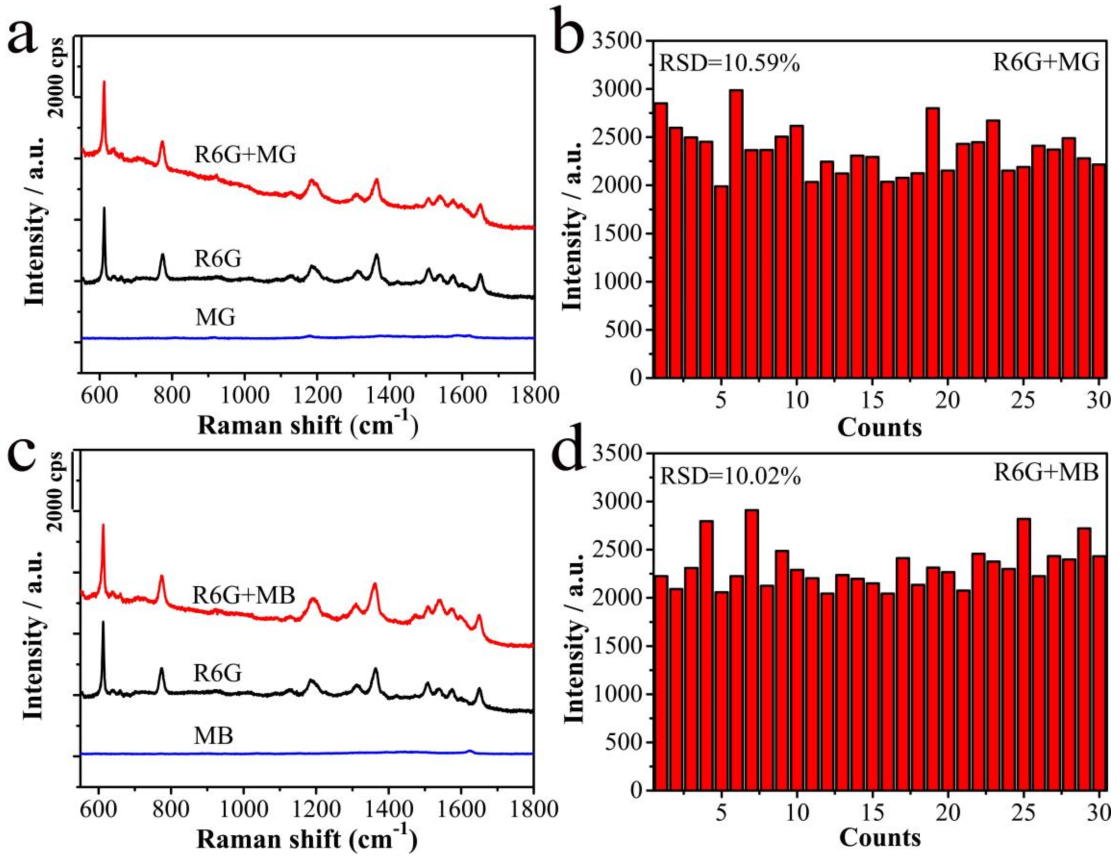

Supplementary Figure 15 Sensitivity to contamination of the SERS substrate. SERS performances of partially-oxidized samples obtained at 300 ℃ for 40 mins upon contamination with (a) methyl green (MG) or (c) methylene blue (MB). Another major concern in using SERS substrate is whether there will be interferences caused by the interaction of the contaminants. R6G-MG and R6G-MB solutions are prepared as model systems by mixing 1 mL of the 1×10^{-4} M solution of R6G with 9 mL of the 1×10^{-5} M solution of MG or MB solution. (a) shows the SERS spectra of R6G, MG and R6G-MG mixture, while (c) shows the SERS spectra of R6G, MB and R6G-MB mixture. As evident from these figures, the SERS spectrum of R6G seems not to be inhibited by the presence of MG or MB, showing good selectivity and sensitivity for

R6G detection.

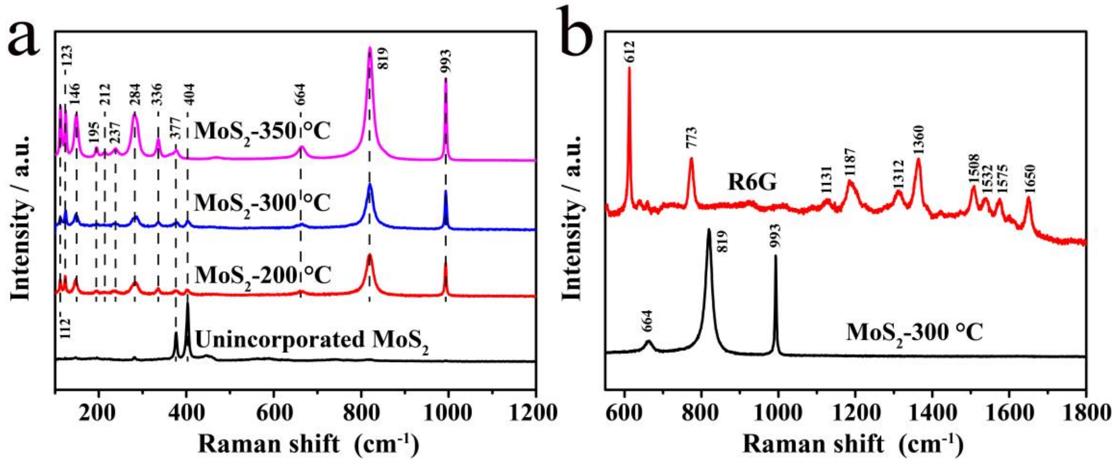

Supplementary Figure 16 Raman spectra of R6G, pristine MoS_2 without R6G loading and partially-oxidized MoS_2 samples without R6G loading. MoS_2 structure can be confirmed by the appearance of two distinct peaks at 377 and 404 cm^{-1} for the E_{2g}^{1} and A_{1g} vibrational modes, respectively[1]. In contrast, partially-oxidized MoS_2 samples show dramatically altered Raman spectra with several additional bands when compared to pristine MoS_2, indicating the successful incorporation of oxygen atoms. Besides the characteristic bands at 377 and 404 cm^{-1} for MoS_2, the bands at 112, 123, 146, 195 cm^{-1} can be ascribed to MoO_2 [2], and the bands at 212, 237, 336 cm^{-1} are related to MoO_3 [3]. The broad band at 284 cm^{-1} (B_{2g}, B_{3g}) is a doublet comprised of wagging modes of the terminal oxygen atoms, while the 664 cm^{-1} (B_{2g}, B_{3g}) is an asymmetric stretching of the Mo–O–Mo bridge along the c axis, and the intense band at 819 cm^{-1} (A_g, B_{1g}) is a symmetric stretch of the terminal oxygen atoms, with another intense band at 993 cm^{-1} (A_g, B_{1g}) being the asymmetric stretch of the terminal oxygen atoms[4]. It is found that the relative intensities of the Raman bands of these oxygen-incorporated samples could vary significantly as a function of treatment temperature; however, there is no frequency shift on these bands. It is also important to note that the Raman bands of the samples at low-temperature treatment (MoS_2-200 ℃ and MoS_2-300 ℃) indicate the coexistence of Mo-S and Mo-O bonds, while for MoS_2-350 ℃ sample after high-temperature treatment, the disappearance of A_{1g} (405 cm^{-1}) band evidences a

decreased amount of S component as well as the phase transition from pristine MoS_2 towards fully-oxidized MoO_3.

Supplementary Tables

Supplementary Table 1 Geometry information of oxygen-incorporated and pristine MoS_2. In-plane lattice constant, interplanar distances of (100) and (001) are denoted by a, d_1 and d_2, respectively. (units: Å)

Å	MoS_2	MoS_xO_y (hollow)	MoS_xO_y (bridge)	Experiment	
				MoS_2	MoS_xO_y
a	3.21	3.13	3.27	----	----
d_1	2.77	2.71	2.83	2.72	2.65
d_2	6.22	6.31	7.65	6.20	6.27

Supplementary Table 2 Elemental analyses of various oxygen-incorporated MoS_2 samples. On the basis of the elemental analyses, it can be seen that there is a relative increase in oxygen concentration in MoS_2 as the annealing temperature increases. The oxygen incorporation concentration increases from 44.81% to 89.64% when the annealing temperature is raised from 250 to 350 ℃.

Atom%	Mo	S	O
200 °C	22.89	52.38	24.73
250 °C	16.37	38.82	44.81
300 °C	16.51	20.76	62.73
350 °C	8.56	1.8	89.64
400 °C	24.85	0	75.15

Supplementary Table 3 Raman Bands for R6G.[4] The Raman frequencies at 612, 773, 1131 cm^{-1} can be assigned to C-C-C ring in-plane, C-H out-of-plane and C-H in-plane bending modes, respectively. The frequencies at 1187 and 1312 cm^{-1} can be

assigned to C-C stretching modes, while the frequencies at 1360, 1508, 1532, 1575 and 1650 cm^{-1} are related with aromatic C-C stretching modes.

Frequency cm^{-1}	Assignment
612	C-C-C ring in-plane bend
773	C-H out-of-plane bend
1131	C-H in-plane bend
1187	C-C stretching
1312	stretching modes
1360	aromatic C-C stretching
1508	aromatic C-C stretching
1532	aromatic C-C stretching
1575	aromatic C-C stretching
1650	aromatic C-C stretching

Supplementary Notes

Supplementary Note 1 Atomic structure of oxygen-incorporated MoS_2:

Two rotational geometries are considered for oxygen-incorporated MoS_2, as shown in supplementary Fig. 1, denoted bridge and hollow configuration. The configurations could be used to describe the arrangement of oxygen atoms with respect to the structure of MoS_2. In the bridge configuration, oxygen atoms sit in the middle of the two sulfur atoms of the two adjacent MoS_2 layers. In contrast, oxygen atoms sit at the center of a hexagon of MoS_2 for hollow configuration. The structures of the two configurations are optimized to achieve the preset convergence criterion, and the corresponding optimized geometry parameters of is summarized in supplementary Table 1.

Supplementary Note 2 Herzberg−Teller coupling term[5]

A-Terms. Molecule to Semiconductor Charge Transfer

$$R_{ICK}(\omega) = \frac{\mu_{KI}\mu_{IC}h_{CK}\langle i|Q_K|f\rangle}{((\varepsilon_1(\omega) + 2\varepsilon_0)^2 + \varepsilon_2^2(\omega))((\omega_{IC}^2-\omega^2) + \gamma_{IC}^2)((\omega_{kI}^2-\omega^2) + \gamma_{KI}^2)} \quad (1)$$

$$R_{ICV}(\omega) = \frac{\mu_{VC}\mu_{IC}h_{IV}\langle i|Q_K|f\rangle}{((\varepsilon_1(\omega) + 2\varepsilon_0)^2 + \varepsilon_2^2(\omega))((\omega_{IC}^2-\omega^2) + \gamma_{IC}^2)((\omega_{VC}^2-\omega^2) + \gamma_{VC}^2)} \quad (2)$$

B-Terms. Semiconductor to Molecule Charge Transfer

$$R_{IVK}(\omega) = \frac{\mu_{VK}\mu_{KI}h_{IV}\langle i|Q_K|f\rangle}{((\varepsilon_1(\omega) + 2\varepsilon_0)^2 + \varepsilon_2^2(\omega))((\omega_{VK}^2-\omega^2) + \gamma_{VK}^2)((\omega_{kI}^2-\omega^2) + \gamma_{KI}^2)} \quad (3)$$

$$R_{KVC}(\omega) = \frac{\mu_{CV}\mu_{VK}h_{KC}\langle i|Q_K|f\rangle}{((\varepsilon_1(\omega) + 2\varepsilon_0)^2 + \varepsilon_2^2(\omega))((\omega_{VK}^2-\omega^2) + \gamma_{VK}^2)((\omega_{CV}^2-\omega^2) + \gamma_{CV}^2)} \quad (4)$$

Supplementary Discussion

The calculation results indicate that the existence of oxygen makes the interplanar spacing of (100) plane vary from 2.77 Å to 2.70 Å and 2.83 Å for hollow and bridge structure, respectively. Since a 2.2% reduction in the interplanar spacing of (100) plane is observed upon oxygen incorporation in experiment, we are convinced that hollow atomic structure is in the proper geometric configuration.

Supplementary Methods

Synthesis of oxygen-substituted MoS_2.

MoS_2 was synthesized according to the reported literature[6]. In brief, 1 mmol $(NH_4)_6Mo_7O_{24}\cdot 4H_2O$ and 30 mmol thiourea were dissolved in 40 mL distilled water under vigorous stirring for 30 min to form a homogeneous solution. Then the above solution was transferred into a 100 mL Teflon-lined stainless-steel autoclave, treated at 200 ℃ for 20 h and cooled down naturally. The black precipitates formed at the

end of the reaction were collected by centrifugation, washed with deionized water and ethanol several times, and finally dried under vacuum at 60 ℃ for 12 h.

Synthesis of oxidized MoS_2.

For partially-oxidized MoS_2, the preparation was achieved through controllably annealing of MoS_2 samples at the different temperatures and times in air. Different annealing times were used from 10 min to 3 hours, while four different annealing temperatures (250, 300, 350, 400 ℃) were designed. A ramp rate of 10 ℃ / min was used for the temperature adjustment. Finally, the oxidized MoS_2 samples with various degrees of oxidation were obtained.

Raman measurement.

Raman spectra of R6G molecule deposited on oxygen-incorporated MoS_2 samples as substrates were obtained under laser excitation at 532.8 nm. Specifically, R6G aqueous solutions with concentration varied from 10^{-4} to 10^{-7} M were obtained from a stock solution of 10^{-3} M by successive dilution. Then1 mL of R6G solution with given concentration was combined with 500 μL of oxygen-incorporated MoS_2 aqueous dispersion (0.5 mg/mL) followed by 2 hours storage in dark to reach the adsorption equilibrium. At last, 20 μL suspension was extracted and dropped onto a cleaned silicon wafer before drying at 60 ^{o}C for at least 2h.

Raman spectra were subsequently acquired on a high-resolution confocal Raman spectrometer (LabRAM HR-800). The spectra were collected by using a 50×L objective lens for 15 s with a laser spot diameter of about 1 μm and power of 0.3 mW in all acquisitions. Raman spectra from different locations were collected for each sample, with the signal intensity averaged for final analysis to estimate the RSD values for enhancement factors.

Calculation of the enhancement factor.

The EF was calculated according to the formula:

$$EF = (I_{SERS}/N_{SERS})/(I_{bulk}/N_{bulk}) \qquad 5$$

$$N_{SERS} = CVN_A A_{Raman}/A_{sub} \qquad 6$$

$$N_{bulk} = \rho h A_{Raman} N_A/M \qquad 7$$

N_{SERS} and N_{bulk} denote the number of R6G molecules that contribute to the signal intensity, enhanced and normal, respectively, while I_{SERS} and I_{bulk} denote the corresponding enhanced and normal Raman intensities. For analyte molecules loaded with SERS-active substrates, N_{SERS} can be estimated by equation 6, assuming that the analyte were distributed uniformly on the surface of substrates. C is the molar concentration of the analyte solution, V is the volume of the droplet, N_A is Avogadro constant. A_{Raman} is the laser spot area (1 μm in diameter) of Raman scanning. Twenty microliters of the droplet on the substrate was spread into a circle of about 3 mm in diameter after solvent evaporation, from which the effective area of the substrate, A_{Sub}, can be obtained. The data for bulk R6G crystals on bare Si/SiO_2 wafer were used as normal Raman reference. The confocal depth (h) of the laser beam into bulk crystal is 21 μm[1], and on the basis of molecular weight (M) and density (ρ) of bulk R6G (1.15 g cm^{-3}), N_{bulk} is calculated by equation 7.

Theoretical Calculation.

The optimize geometries and electronic properties of pristine MoS_2 and oxygen-incorporated MoS_2 were calculated at the density functional theory (DFT) level as implemented in the Vienna ab initio simulation package (VASP)[7], using the projector-augmented wave (PAW) potentials with a plane-wave cutoff of 600 eV[8]. The Perdew-Burke-Ernzerhof (PBE) form of the exchange-correlations functional was employed in the simulation[9], and the long-range interlayer van der Waals (vdW) interaction was described with DFT-D2 method[10]. The crystal structures were simulated by a primitive cell with a $15\times15\times5$ k-point mesh to sample the Brillouin zone, and optimized by using the conjugate gradient method, in which the convergence for total energy and interaction force was set to be 10^{-6} eV and 10^{-3} eV/Å, respectively. In order to circumvent the well-known inaccuracy of DFT in predicting band structures, the GW method was performed to obtain more accurate band energies.

Supplementary References

1. Kibsgaard, J., Chen, Z., Reinecke, B. N. & Jaramillo, T. F. Engineering the surface structure of MoS_2 to preferentially expose active edge sites for electrocatalysis. *Nat. Mater.* **11**, 963-969 (2012).
2. Zhang, Q et al. A metallic molybdenum dioxide with high stability for surface enhanced Raman spectroscopy. *Nat. Commun.* **8**, 14903 (2017).
3. Windom, B. C., Sawyer, W. G. & Hahn, D. W. A Raman spectroscopic study of MoS_2 and MoO_3: applications to tribological systems. *Tribol. Lett.* **42** 301–310 (2011).
4. Hildebrandt, P. & Stockburger, M. Surface-enhanced resonance Raman spectroscopy of Rhodamine 6G adsorbed on colloidal silver. *J. Phys. Chem.* **88**, 5935-5944 (1984).
5. Lombardi, J. R. & Birke, R. L. Theory of surface-enhanced Raman scattering in semiconductors. *J. Phys. Chem. C* **118**, 11120-11130 (2014).
6. Xie, J. et al. Controllable disorder engineering in oxygen-incorporated MoS_2 ultrathin nanosheets for efficient hydrogen evolution. *J. Am. Chem. Soc.* **135**, 17881-17888 (2013).
7. Kresse, G. & Furthmüller, J. Efficient iterative schemes for ab initio total-energy calculations using a plane-wave basis set. *Phys. Rev. B* **54**, 11169 (1996).
8. Blöchl, P. E. Projector augmented-wave method. *Phys. Rev. B* **50**, 17953 (1994).
9. Perdew, J. P., Burke, K. & Ernzerhof, M. Generalized gradient approximation made simple. *Phys. Rev. Lett.* **77**, 3865 (1996).
10. Grimme, S. Semiempirical GGA-type density functional constructed with a long-range dispersion correction. *J. Comp. Chem.* **27**, 1787-1799 (2006).
